# Intravenous Vitamin C for Cancer Therapy – Identifying the Current Gaps in Our Knowledge

**DOI:** 10.3389/fphys.2018.01182

**Published:** 2018-08-23

**Authors:** Anitra C. Carr, John Cook

**Affiliations:** ^1^Department of Pathology and Biomedical Science, University of Otago, Christchurch, New Zealand; ^2^New Brighton Health Care, Christchurch, New Zealand

**Keywords:** vitamin C, ascorbate, intravenous vitamin C, cancer, chemotherapy, pharmacokinetics, enzyme cofactor, quality of life

## Abstract

The use of intravenous vitamin C (IVC) for cancer therapy has long been an area of intense controversy. Despite this, high dose IVC has been administered for decades by complementary health care practitioners and physicians, with little evidence base resulting in inconsistent clinical practice. In this review we pose a series of questions of relevance to both researchers and clinicians, and also patients themselves, in order to identify current gaps in our knowledge. These questions include: Do oncology patients have compromised vitamin C status? Is intravenous the optimal route of vitamin C administration? Is IVC safe? Does IVC interfere with chemotherapy or radiotherapy? Does IVC decrease the toxic side effects of chemotherapy and improve quality of life? What are the relevant mechanisms of action of IVC? What are the optimal doses, frequency, and duration of IVC therapy? Researchers have made massive strides over the last 20 years and have addressed many of these important aspects, such as the best route for administration, safety, interactions with chemotherapy, quality of life, and potential mechanisms of action. However, we still do not know the answers to a number of fundamental questions around best clinical practice, such as how much, how often and for how long to administer IVC to oncology patients. These questions point the way forward for both basic research and future clinical trials.

## Introduction

Over the years, numerous epidemiological studies have highlighted a decreased incidence of cancer and improved survival in patients with higher dietary intakes of vitamin C or higher plasma levels of the vitamin (Carr and Frei, 1999b; Harris et al., 2014). Although vitamin C is often considered a good marker of fruit and vegetable intake (Block et al., 2001), the vitamin has essential functions within the body, including integral roles in various anti-cancer mechanisms (Du et al., 2012). Because of the pleiotropic functions of vitamin C, optimizing its levels in the body through diet and supplementation is likely to be of benefit to oncology patients. In support of this premise, studies in a vitamin C-requiring mouse model indicate that oral vitamin C supplementation of these animals can impair the development of tumors and can also increase the rejection rate of implanted tumor cells (Campbell et al., 2016a; Cha et al., 2016). This suggests an important role for vitamin C in host defense against cancer.

Treatment of cancer, in contrast, is thought to require much higher doses of vitamin C than normal dietary intakes (Parrow et al., 2013). In fact, high dose intravenous vitamin C (IVC) has been administered by physicians for many decades as a complementary and alternative therapy for oncology patients (Padayatty et al., 2010). This practice has continued despite significant controversy in the field as a result of two high profile Mayo Clinic trials carried out in the late 1970s which debunked the initially encouraging findings of Cameron and Pauling (1976, 1978; Creagan et al., 1979; Moertel et al., 1985). Due to the paucity of a strong evidence base for informing appropriate clinical practice, there are significant inconsistencies in administration of IVC therapy to oncology patients (Padayatty et al., 2010). These include huge variability in the dose, frequency, and duration of vitamin C administration.

Research in the 1990s highlighting the important differences between oral and IVC pharmacokinetics resulted in a surge of new research in the IVC field with numerous *in vitro*, preclinical and clinical studies being undertaken (Parrow et al., 2013; Fritz et al., 2014). The *in vitro* studies have provided useful insights into potential mechanisms of action and pre-clinical studies have indicated promising efficacy of IVC, however, clinical studies have so far been limited primarily to Phase I safety and pharmacokinetic trials (Du et al., 2012; Parrow et al., 2013). As a result of study design issues with many of the earlier clinical trials there remains controversy as to the efficacy of IVC in the treatment of cancer (Wilson et al., 2014). Many of these trials recruited terminal or end-stage patients, for whom any sort of treatment is unlikely to have an effect, and they often recruited cohorts with mixed cancers, which may respond differentially to IVC depending on the underlying mechanisms involved.

In this review, we pose a series of questions, both clinically relevant and patient centered, related to IVC use in cancer therapy in order to identify the current gaps in our knowledge. Although many of these questions have been adequately addressed, some require further research in order to provide the essential data required to inform good clinical practice.

## Q1. Do Oncology Patients Have Compromised Vitamin C Status?

Vitamin C supports many important biological functions through its action as an electron donor (Du et al., 2012), however, the vitamin C status of oncology patients is often not assessed in clinical trials or in clinical practice. Many studies (shown in **Table 1**) have consistently shown that patients with cancer have lower mean plasma vitamin C status than healthy controls (Torun et al., 1995; Choi et al., 1999; Mahdavi et al., 2009; Sharma et al., 2009; Emri et al., 2012; Mehdi et al., 2013; Huijskens et al., 2016), and a large proportion of them present with hypovitaminosis C (<23 μmol/L) and outright deficiency (<11 μmol/L) (Anthony and Schorah, 1982; Fain et al., 1998; Mayland et al., 2005; Riordan et al., 2005; Hoffer et al., 2015; Liu et al., 2016; Shenoy et al., 2017). Although other case control studies have confirmed lower vitamin C status in patients with cancer (Khanzode et al., 2003; Gupta et al., 2009), their control values were also quite low indicating possible issues with sample collection and/or analysis (Pullar et al., 2018). Severity of the disease also appears to impact on vitamin C status with the proportion of lymphoma patients with hypovitaminosis C being significantly elevated for those with high-burden disease (Shenoy et al., 2017). Furthermore, patients with higher stage breast and cervical cancers had significantly lower vitamin C status than patients with lower stage disease (Ramaswamy and Krishnamoorthy, 1996; Khanzode et al., 2004). *Ex vivo* studies indicate that tumors from patients with colorectal cancer contained lower vitamin C concentrations than matched normal tissue (Kuiper et al., 2014a), and higher grade colorectal and endometrial tumors had proportionately less vitamin C than lower grade tumors (Kuiper et al., 2010, 2014a).

**Table 1:** Vitamin C levels in plasma or serum of oncology patients.

Study types	Study cohorts	Vitamin C levels	Reference
Case control	22 controls 57 patients with cancer (gastrointestinal, head and neck, lung)	Controls: 51 μmol/L Cases: 10 μmol/L^∗∗^	Mahdavi et al., 2009
Case control	156 controls 208 patients with cancer (breast, head and neck, genitourinary, lung, gastrointestinal, etc.)	Controls: 50 μmol/L Cases: 23 μmol/L^∗^	Torun et al., 1995
Case control	50 controls 50 patients with multiple myeloma	Controls: 81 μmol/L Cases: 30 μmol/L^∗∗^	Sharma et al., 2009
Case control	30 controls 30 patients with multiple myeloma	Controls: 52 μmol/L Cases: 39 μmol/L^∗∗^	Mehdi et al., 2013
Case control	83 controls 42 patients with malignant mesothelioma	Controls: 49 μmol/L Cases: 30 μmol/L^∗∗^	Emri et al., 2012
Case control	76 controls 42 patients with hematological malignancies	Controls: 65 μmol/L Cases: 21 μmol/L^∗∗∗^ 19% <11 μmol/L	Huijskens et al., 2016
Single arm	34 patients with lymphoma 14 high burden disease 20 low burden disease	29% <23 μmol/L 64% <23 μmol/L^∗∗^ 5% <23 μmol/L	Shenoy et al., 2017
Single arm	50 patients with cancer (brain, breast, bronchial, urogenital, prostate, gastrointestinal, etc.)	72% <23 μmol/L 30% <11 μmol/L	Mayland et al., 2005
Single arm	22 patients with cancer (mostly colon)	73% <50 μmol/L 60% <23 μmol/L 50% <11 μmol/L	Riordan et al., 2005
Single arm	12 patients with cancer (mostly colon and rectum)	67% <50 μmol/L 8% <11 μmol/L	Hoffer et al., 2015
Single arm	24 patients with hematopoietic cancers	92% <26 μmol/L 58% <11 μmol/L	Liu et al., 2016
Single arm	139 patients with lung cancer	70% <20 μmol/L 30% <6 μmol/L	Anthony and Schorah, 1982

*^∗^P < 0.05; ^∗∗^P < 0.001; ^∗∗∗^P < 0.0001. <50 μmol/L inadequate, <23 μmol/L hypovitaminosis C, <11 μmol/L deficient and at risk of developing scurvy (Lykkesfeldt and Poulsen, 2010).*

This observational data supports the premise that oncology patients have compromised vitamin C status, which is likely due to enhanced metabolic turnover as a result of oxidative and inflammatory aspects of the disease process (Reuter et al., 2010). Enhanced oxidative stress and pro-inflammatory biomarkers can also result from chemotherapy (Hunnisett et al., 1995; Nannya et al., 2014). Administration of IVC to oncology patients results in lower circulating vitamin C levels compared with administration of the same amount to healthy controls (Mikirova et al., 2013). The finding that oncology patients have a higher requirement for vitamin C is thought to indicate a lower body pool and/or the higher oxidative and pro-inflammatory status of these patients, as indicated by elevated lipid oxidation products or C-reactive protein concentrations (Torun et al., 1995; Mayland et al., 2005; Mahdavi et al., 2009; Sharma et al., 2009; Mehdi et al., 2013; Mikirova et al., 2013). It is interesting to note that animals which can synthesize their own vitamin C in their livers increase their endogenous vitamin C levels when under a tumor burden suggesting enhanced requirements (Campbell et al., 2015; Cha et al., 2016).

There is evidence that adjunctive cancer therapies may impact negatively on vitamin C status (**Table 2**). It has been noted that administration of some anticancer therapies, such as cisplatin, fluorouracil, nilotinib, and interleukin-2, can significantly lower the vitamin C status of oncology patients and result in scurvy-like symptoms in some cases (Marcus et al., 1991; Fain et al., 1998; Weijl et al., 1998; Alexandrescu et al., 2009; Oak et al., 2016). Chemotherapy drugs such as cisplatin are known to generate off-target oxidative stress which could contribute to the depletion of vitamin C (Chirino and Pedraza-Chaverri, 2009). Discontinuation of the chemotherapy or administration of supplemental vitamin C to these patients resolved the deficiency symptoms (Fain et al., 1998; Alexandrescu et al., 2009; Oak et al., 2016). Patients with hematopoietic cancers typically undergo conditioning with multiple chemotherapy agents and a number of studies have shown significantly lower vitamin C status following the conditioning regimens (Hunnisett et al., 1995; Goncalves et al., 2009; Nannya et al., 2014; Huijskens et al., 2016; Liu et al., 2016; Rasheed et al., 2017). Although plasma vitamin C levels appear to return to baseline values approximately 1 month following chemotherapy (Mehdi et al., 2013; Rasheed et al., 2017), these values are still well below optimal. Administration of IVC to these patients has been shown to increase their circulating vitamin C levels and decrease markers of lipid oxidation (Hunnisett et al., 1995; Jonas et al., 2000).

**Table 2:** Effects of chemotherapeutic agents on plasma vitamin C levels in oncology patients.

Study types	Study cohorts	Vitamin C patients. levels	Reference
Single arm	34 patients with cancer (osteosarcoma, testicular) + cisplatin combinations	Before chemo: 46 μmol/L After chemo: 33 μmol/L^∗∗^	Weijl et al., 1998
Single arm	15 patients with cancer (melanoma, colon, kidney) + interleukin-2	Before chemo: 36 μmol/L After chemo: 7 μmol/L^∗∗∗^	Marcus et al., 1991
Single arm	24 patients with hematopoietic cancers (bone marrow transplant) + chemotherapy	Before chemo: 48 μmol/L After chemo: 49 μmol/L	Jonas et al., 2000
Single arm	20 patients with hematopoietic cancers (bone marrow transplant) + chemotherapy and/or total body irradiation	Before: 34 or 43 μmol/L After: 14 or 15 μmol/L^∗∗^	Hunnisett et al., 1995
Single arm	15 patients with hematopoietic cancers (stem cell transplantation) + conditioning regimens	Before chemo: 37 μmol/L After chemo: 22 μmol/L^#^ At day 14: 12 μmol/L^#^	Nannya et al., 2014
Single arm	15 patients with hematopoietic cancers (stem cell transplantation) + conditioning regimens	Before chemo: 41 μmol/L After chemo: 27 μmol/L^∗^ At day 14: 22 μmol/L^∗^ At day 30: 34 μmol/L	Rasheed et al., 2017
Case control	30 patients with multiple myeloma before treatment 30 patients with multiple myeloma 1 month after chemotherapy	Before chemo: 39 μmol/L After 1 month: 42 μmol/L^∗^	Mehdi et al., 2013

*^∗^P < 0.05; ^∗∗^P < 0.001; ^∗∗∗^P < 0.0001, ^#^P-value not reported.*

## Q2. Is IV the Optimal Route for Vitamin C Administration?

Much of the controversy around vitamin C use in cancer therapy has arisen from early misunderstandings around the pharmacokinetics of oral and IVC. In the mid 1970s Cameron and Pauling (1976, 1978) published findings from 100 terminal cancer patients who had been administered 10 g/d IVC for approximately 10 days, followed by 10 g/d oral vitamin C thereafter. Their work showed significantly enhanced survival in these patients compared with two retrospective cohorts of 1,000 patients who did not receive vitamin C. Subsequently, researchers at the Mayo Clinic attempted to reproduce these results with two randomized controlled trials (RCTs) carried out in 123 patients with advanced malignancies and 100 patients with advanced colorectal cancer (Creagan et al., 1979; Moertel et al., 1985). Neither study showed a significant survival advantage in the patients who received vitamin C.

The negative results of the Mayo Clinic RCTs relegated the use of vitamin C to the arena of complementary and alternative medicine (Padayatty et al., 2010). It wasn’t until the mid 1990s, when the seminal work by Mark Levine’s group highlighted the dramatic differences between the pharmacokinetics of oral and IVC, that the discrepancies between the original Cameron and Pauling studies and the Mayo Clinic RCTs were explained (Levine et al., 1996). While Cameron and Pauling (1976, 1978) had used IVC with subsequent oral maintenance, the Mayo Clinic RCTs comprised divided daily doses of oral vitamin C only (Creagan et al., 1979; Moertel et al., 1985). Intestinal uptake of oral vitamin C is regulated via the sodium-dependent vitamin C transporter-1 (SVCT1) (Savini et al., 2008), which is bypassed with IVC administration, resulting in significantly higher plasma concentrations (Levine et al., 1996). Peak plasma concentrations from oral ingestion rarely exceed 200 μmol/L (Padayatty et al., 2004), however, IVC administration can result in peak plasma concentrations of 20 mmol/L (**Table 3**). These high concentrations are relatively transient, however, due to rapid clearance by the kidneys, resulting in a half-life of about 2 h in circulation (Stephenson et al., 2013; Nielsen et al., 2015). The realization that IVC may be more effective than oral vitamin C in oncology patients sparked a surge of new research in the field over the past 20 years, particularly around plausible mechanisms of action (Parrow et al., 2013; Fritz et al., 2014).

**Table 3:** Pharmacokinetics of high dose vitamin C in preclinical cancer models and oncology patients with and without chemotherapy.

Model/Cancer	IVC dose (g/kg)^a^	*C*_max_ (mM)	AUC (mM^∗^h)	*t*_1/2_ (h)	Reference
**Preclinical studies**					
Gulo^−/−^ mice, Lewis lung (LL/2)	1.0 (IV)	5	5	1.3	Campbell et al., 2016b
Nude mice, hepatoma (TLT)	1.0 (IP) 1.0 (IV)	7 22	12 20	1.0 0.7	Verrax and Calderon, 2009
Wistar rats, no tumors	0.5 (IP) 0.5 (IV)	3 8	– –	– –	Chen et al., 2007
**Without chemotherapy**					
12 advanced cancer	0.6	14	–	–	Hoffer et al., 2015
10 prostate cancer	0.07–0.7 (5–60 g/d)	2–20	4–48	1.7–2.0	Nielsen et al., 2015
15 advanced cancer	0.8–3.0 (30–110 g/m^2^)	23–37	74–217	2.1–2.5	Stephenson et al., 2013
11 advanced cancer	0.1–1.5	2.4–26	6–93^b^	–	Hoffer et al., 2008
**With chemotherapy**					
12 advanced cancer with chemotherapy	0.6	14	–	–	Hoffer et al., 2015
9 pancreatic cancer with gemcitabine	0.2–1.8 (15–125 g/d)	5–30	–	–	Welsh et al., 2013
25 ovarian cancer with carboplatin, paclitaxel	1.1–1.4 (75–100 g/d)	20–23	–	–	Ma et al., 2014
14 pancreatic cancer with gemcitabine, erlotinib	1.1–1.4 (75–100 g/d)	20–30	–	–	Monti et al., 2012

*^a^Doses not reported in g/kg were calculated assuming 70 kg person or g/m^2^ = g/kg^∗^37. ^b^AUC calculated by multiplying dose in g/kg by 62. C_max_, maximum plasma concentration; t_1/2_, half-life; AUC, area under the concentration time curve; IV, intravenous; IP, intraperitoneal.*

## Q3. Is IVC Safe?

High dose IVC has been used for many decades by complementary and alternative medicine providers and physicians, with few side effects reported (Padayatty et al., 2010). Of 9,328 patients surveyed, only 1% reported minor side effects that included lethargy, fatigue, change in mental status and vein irritation. More recent Phase 1 safety trials of high dose IVC indicate only minor side effects and no adverse events over and above what would be expected from the underlying disease or chemotherapy side effects (Hoffer et al., 2008; Monti et al., 2012; Stephenson et al., 2013; Welsh et al., 2013).

A minor product of vitamin C metabolism is oxalate which has the potential to form calcium oxalate crystals in individuals predisposed to renal stone formation. One patient with a history of renal calculi developed a kidney stone following 2 weeks of continuous IVC infusion (Riordan et al., 2005). Acute oxalate nephropathy has also been reported in several cases following high dose IVC administration, however, the patients all exhibited existing renal dysfunction (Lawton et al., 1985; Wong et al., 1994; Cossey et al., 2013). Therefore, high dose IVC is contraindicated for patients with renal dysfunction due to the inability of the kidneys to clear high circulating concentrations. However, in individuals with normal renal function, IVC infusions of up to 1.5 g/kg body weight resulted in less than 0.5% conversion into oxalic acid (Robitaille et al., 2009).

Glucose-6-phosphate dehydrogenase (G6PD) deficiency is typically screened for prior to high dose IVC administration, due to two case reports of hemolytic anemia in G6PD deficient individuals following 80 g IVC administration (Rees et al., 1993; Quinn et al., 2017). The lower IVC doses typically used for quality of life improvement (e.g., ≤10 g/d) would be unlikely to precipitate hemolytic anemia in G6PD deficient individuals due to lack of *in vivo* hydrogen peroxide generation at these doses.

Intravenous vitamin C has been shown to interfere with many point-of-care glucose meters, even at low gram doses (Tang et al., 2000). IVC can cause either false positive or false negative results depending on the biochemistry utilized in the monitor, therefore, caution is required for patients needing regular glucose monitoring. However, IVC does not interfere with laboratory-based glucose tests which utilize absorbance photometric rather than electrochemical detection (Tang et al., 2000; Kahn and Lentz, 2015). Some clinicians have used the detection of IVC by point-of-care glucose monitors as a convenient method for determining peak plasma vitamin C concentrations in patients receiving IVC infusions (Ma et al., 2013).

## Q4. Does IVC Interfere With Chemotherapy or Radiotherapy?

Potential interactions between vitamin C and chemotherapeutic agents have long been a matter of debate (D’Andrea, 2005; Simone et al., 2007; Lawenda et al., 2008; Verrax and Calderon, 2008). Because of vitamin C’s potent antioxidant activity, many clinicians believe they have to avoid the concurrent use of IVC during all chemotherapy regimens. However, different chemotherapeutic agents act via different mechanisms, and only some act via oxidative mechanisms (Simone et al., 2007). Nevertheless, it has been recommended for administered natural therapies to allow five half-lives to elapse prior to administration of chemotherapeutic agents to eliminate potential interactions (Seely et al., 2007). Because vitamin C has a relatively short half-life of <2 h in circulation due to rapid renal clearance, IVC is typically administered the day before or after chemotherapy administration (Stephenson et al., 2013; Nielsen et al., 2015). However, if the chemotherapeutic agent does not act via oxidative mechanisms, then concurrent IVC administration may not be an issue and would be more patient centered.

Animal studies have indicated that concurrent administration of vitamin C to numerous different chemotherapeutic agents (e.g., gemcitabine, paclitaxel, carboplatin, melphalan, carfilzomib, bortezomib, cisplatin, and temozolomide) synergistically decreased xenograft tumor growth, including in a chemotherapy resistant pancreatic tumor model, and synergistically increased survival (**Table 4**) (Espey et al., 2011; Ma et al., 2014; Wang C. et al., 2017; Xia et al., 2017). No difference in the anti-tumor activity of the chemotherapeutic agents dacarbazine and valproic acid was observed when combined with high dose parenteral vitamin C in a murine melanoma model (Serrano et al., 2015). Administration of vitamin C to mice and guinea pigs reduced the toxic side effects of paclitaxel and doxorubicin without interfering with their antitumor effects (Fujita et al., 1982; Park et al., 2012). Interestingly, many of the pre-clinical studies showed that administration of IVC alone was as effective at decreasing tumor growth and promoting survival as the chemotherapeutic agents themselves (**Table 4**).

**Table 4:** Summary of the effects of high-dose vitamin C administration in pre-clinical cancer models.

Treatments	Animal models	Tumor types	Findings	Reference
**Vitamin C administered alone**				
Oral vitamin C 0.15–5 g/L or <0.5 g/kg/d	Murine (SCID, C57BL/6, Gulo^−/−^) or guinea pig	Melanoma (B16F10), Lewis lung (LL/2), lymphoma (P493), colorectal (CMT-93), breast (4T1), liver (L-10)	Increased plasma and tumor vitamin C levels Decreased tumor development and growth Increased collagen encapsulation and decreased metastasis Decreased pro-survival protein levels (e.g., HIF-1α, GLUT-1, VEGF) Decreased pro-inflammatory cytokines (e.g., IL-6, IL-1β)	Casciari et al., 2005; Cha et al., 2011, 2013, 2016; Gao et al., 2007; Campbell et al., 2015, 2016a; Yang et al., 2017)
IV or IP vitamin C 0.1–8 g/kg/d for 2–4 weeks	Murine (wild type, nude, SCID, Gulo^−/−^)	Colon (HT29, CT26, WiDr), ovarian (Ovar5), pancreatic (Pan02, MIA PaCa-2), glioblastoma (9L), hepatoma (TLT), lung (H322, LL/2), sarcoma (S180), prostate (PAIII), colorectal (HCT116, VACO432)	Increased plasma and tumor vitamin C levels Decreased tumor growth and decreased metastasis Increased survival Generation of ROS (e.g., H_2_O_2,_ ascorbyl radicals) Altered gene expression (e.g., VEGF, bFGF, MMP2, cell cycle progression genes) Altered protein levels (e.g., HIF-1α, GLUT-1)	Chen et al., 2008; Belin et al., 2009; Verrax and Calderon, 2009; Yeom et al., 2009; Du et al., 2010; Pollard et al., 2010; Lee et al., 2012; Mamede et al., 2012; Yun et al., 2015; Campbell et al., 2016b; Wilkes et al., 2018
**Vitamin C administered with thiol antioxidants**				
IP vitamin C 4–8 g/kg/d with GSH or NAC	Murine (nude)	Pancreatic (Pan02), colorectal (HCT116)	High dose GSH or NAC inhibited vitamin C-dependent decrease in tumor growth	Chen et al., 2011; Yun et al., 2015
**Vitamin C administered with chemotherapy**				
IV or IP vitamin C 0.15–6 g/kg/d with gemcitabine, paclitaxel, carboplatin, doxorubicin, dacarbazine, valproic acid, chlorambucil, melphalan, carfilzomib, bortezomib, cisplatin	Murine (wild type, nude, NOD, SCID)	Pancreatic (Pan02, PANC-1), sarcoma (S180), leukemia (L1210), melanoma (B16-F10), lymphoma, ovarian (SHIN3)	Decreased tumor growth (synergistic and non-synergistic) Increased survival (synergistic) Decreased off-target toxicity (e.g., decreased: leukocyte loss, reticulocytosis, weight loss, ascites accumulation, hepatotoxicity, cardiomyopathy, renal toxicity, lipid oxidation) Decreased protein levels synergistically (e.g., VEGF, bFGF, mTOR)	Fujita et al., 1982; Espey et al., 2011; Park et al., 2012; Kalita et al., 2014; Ma et al., 2014; Serrano et al., 2015; Wang C. et al., 2017; Xia et al., 2017
**Vitamin C administered with radiation**				
IP vitamin C 4 g/kg/d with radiation	Murine (nude)	Pancreatic (MIA PaCa-2, PANC-1)	Decreased tumor growth and proliferation (synergistic) Increased survival (synergistic) Partially protected against intestinal damage	Du et al., 2015; Cieslak et al., 2016
IP vitamin C 1–2 g/kg/d with radiation	Murine (wild type)	Glioma (GL261)	Administration 2 h prior to radiation attenuated radiation-dependent increase in survival (radio-protective)	Grasso et al., 2014
**Vitamin C administered with radiation and chemotherapy**				
IP vitamin C 4 g/kg/d with radiation and gemcitabine, carboplatin, temozolomide	Murine (nude)	Pancreatic (MIA PaCa-2), non-small-cell lung cancer (H292), glioblastoma (U87)	Decreased tumor growth (synergistic) Increased survival (synergistic)	Cieslak and Cullen, 2015; Schoenfeld et al., 2017

*Only Gulo^−/−^ mice and guinea pigs are vitamin C-requiring animals, all other animal models can synthesize their own vitamin C. DHA, dehydroascorbic acid; GSH, glutathione; IP, intraperitoneal; IV, intravenous; NAC, N-acetylcysteine; ROS, reactive oxygen species; SCID, severe combined immunodeficiency.*

Human trials have shown no adverse effects from combining IVC with a number of different chemotherapeutic agents (e.g., carboplatin, paclitaxel, decitabine, cytarabine, aclarubicin, gemcitabine, erlotinib, and temozolomide; **Table 5**), and in many cases decreased off-target toxicity and improved health-related quality of life were observed (Welsh et al., 2013; Kawada et al., 2014; Ma et al., 2014; Hoffer et al., 2015; Polireddy et al., 2017; Zhao et al., 2018). Vitamin C is routinely administered in combination with arsenic trioxide to enhance its efficacy in the treatment of refractory multiple myeloma (Fritz et al., 2014). Although co-administration of high dose thiol antioxidants, such as glutathione and *N*-acetyl cysteine, was contraindicated in animal models (Chen et al., 2011; Yun et al., 2015), normal supplemental intakes of these antioxidants (e.g., 1–1.5 g/d) by patients would be unlikely to interact with high dose IVC treatments (Padayatty et al., 2006).

**Table 5:** Summary of the effects of IVC administration in clinical studies.

Treatments	Study types	Cancer types	Findings	Reference
**Vitamin C administered alone**				
Oral and/or IVC 0.5–30 g/d daily for duration	Case controls	Advanced cancer	Improved quality of life Decreased need for narcotics Increased survival	Cameron and Campbell, 1974, 1991; Cameron and Pauling, 1976, 1978; Murata et al., 1982
IVC 10–200 g/d one to four times per week for 2–4 weeks	Single arms	Advanced cancer	No serious adverse events Improved quality of life Decreased pro-inflammatory biomarkers	Yeom et al., 2007; Hoffer et al., 2008; Mikirova et al., 2012, 2016; Takahashi et al., 2012; Stephenson et al., 2013
IVC 60 g/d once a week for 12 weeks	Single arm	Prostate cancer	No decrease in PSA No disease remission	Nielsen et al., 2017
**Vitamin C administered with chemotherapy**				
IVC 75–100 g/d two times per week for 12 months	RCT	Ovarian cancer with carboplatin, paclitaxel	Decreased chemotherapy-related organ toxicity and adverse events Minimal effect on survival Minimal change in time to disease relapse/progression	Ma et al., 2014
IVC 3.5–5.6 g/70 kg/d daily for 9 days	RCT	Acute myeloid leukemia with decitabine, cytarabine, aclarubicin	Increased complete remission after first induction Prolonged overall survival Comparable side effects	Zhao et al., 2018
IVC 50–125 g/d two to three times per week for 2 weeks to 18 months	Single arms	Advanced cancer, lymphoma, pancreatic cancer with chemotherapy, or gemcitabine, erlotinib	No serious adverse events Improved quality of life Decreased oxidative stress biomarkers	Monti et al., 2012; Welsh et al., 2013; Kawada et al., 2014; Hoffer et al., 2015; Polireddy et al., 2017
**Vitamin C administered with radiation**				
IVC 2.5 g/d during increasing pain	Case control	Bone metastasis with palliative radiotherapy	Decreased pain Increased survival compared with controls and chemotherapy	Gunes-Bayir and Kiziltan, 2015
**Vitamin C administered with radiation and/or chemotherapy**				
IVC 7.5 g/d once per week for at least 4 weeks	Case control	Breast cancer with radiotherapy and/or chemotherapy	Decreased complaints No adverse side effects	Vollbracht et al., 2011
IVC 75–87.5 g/d two to three times per week for up to 35 weeks	Single arms	Glioblastoma with radiotherapy and temozolomide or non-small-cell lung cancer with carboplatin and paclitaxel	Extended median survival Stable disease or confirmed partial response	Schoenfeld et al., 2017
IVC 15–100 g/d two times per week or less for 12–48 months	Case reports	Advanced cancer with or without radiotherapy and chemotherapy	Regression/resolution of metastases Survival beyond prognosis Good quality of life Decreased chemotherapy side effects No adverse effects	Riordan et al., 2004; Padayatty et al., 2006; Raymond et al., 2016

*IVC, intravenous vitamin C; RCT, randomized controlled trial.*

Radiotherapy with ionizing radiation generates free radicals and also increases levels of catalytic transition metal ions in tissues (Cieslak and Cullen, 2015). Cell culture studies have shown radio-sensitizing effects of high dose vitamin C in combination with ionizing irradiation (Shinozaki et al., 2011; Herst et al., 2012; Hosokawa et al., 2015). Only a few studies have been carried out in animal models, with most showing radio-sensitizing effects of vitamin C (Du et al., 2015; Cieslak et al., 2016), although one model showed radio-protective effects (Grasso et al., 2014). These differences likely reflect the timing of the radiation treatment relative to vitamin C administration. For example, in the Grasso et al. (2014) study, radiation treatment was carried out only 2 h after intraperitoneal administration of vitamin C, while in the other studies radiation treatment was carried out on days 3 or 5 following initiation of high dose vitamin C administration (Du et al., 2015; Cieslak et al., 2016). In these latter studies, high dose vitamin C administration was shown to act synergistically with radiotherapy, decreasing tumor growth and enhancing survival (Cieslak and Cullen, 2015; Cieslak et al., 2016). PET imaging showed tumoristatic activity of vitamin C administration alone and radiation therapy alone, however, the two combined showed tumoricidal activity (Cieslak et al., 2016). Addition of vitamin C to radiotherapy and chemotherapy combinations further improved anti-tumor activity and survival (Cieslak and Cullen, 2015; Schoenfeld et al., 2017). Clearly, more studies need to be carried out to determine appropriate tumor models and optimal timing and dosing of IVC combined with radiotherapy.

## Q5. Does IVC Decrease the Toxic Side Effects of Chemotherapy and Improve Quality of Life?

Numerous animal studies have shown decreased off-target toxicity of chemotherapeutic agents following administration of oral and IVC (**Table 4**). Vitamin C administration decreased white blood cell loss, weight loss, ascites accumulation, hepatotoxicity, reticulocytosis, lipid oxidation, and cardiomyopathy induced by the chemotherapeutic agents. In a murine ovarian cancer model, a synergistic decrease in ascites accumulation was observed with high dose vitamin C administration in combination with carboplatin and paclitaxel (Ma et al., 2014). Administration of vitamin C also decreased the toxic side effects of doxorubicin and paclitaxel, as well as cisplatin, which is known to cause off-target oxidative stress (Fujita et al., 1982; Park et al., 2012; Chen et al., 2014). It has been noted that combination therapy with vitamin C enhances sensitivity to specific anti-cancer drugs, thus potentially decreasing the required dosage and ameliorating associated side-effects (Cimmino et al., 2017; Wang G. et al., 2017). Low dose IVC (e.g., 1 g/infusion) is often administered with arsenic trioxide for multiple myeloma or leukemia to improve tolerability of the chemotherapeutic agent (Fritz et al., 2014).

Decreased chemotherapy-related toxicity has been demonstrated in patients with stage III–IV ovarian cancer receiving chemotherapy (carboplatin and paclitaxel) in conjunction with high-dose IVC (75–100 g two times per week) (Ma et al., 2014). Adverse events were evaluated and grade 1 and 2 toxicities were found to be significantly decreased in the group receiving IVC. Decreased toxicities were observed in almost all the evaluated organ systems, e.g., neurological, bone marrow, hepatobiliary/pancreatic, renal/genitourinary, infection, pulmonary, gastrointestinal, and dermatological. Interestingly, despite decreasing chemotherapy-related toxicity, IVC did not appear to adversely affect the anti-cancer activity of the drugs as assessed by survival of the patients or time to disease relapse or progression (Ma et al., 2014). In support of a role for vitamin C in decreasing chemotherapy-related toxicity, a Phase I clinical study of patients with pancreatic cancer treated with gemcitabine reported decreased plasma F_2_-isoprostanes, an established oxidative stress biomarker, following administration of high dose IVC (Welsh et al., 2013). Thus, IVC is a potentially useful adjunctive therapy to decrease off-target toxicity of chemotherapeutic agents and as a result may also improve the health-related quality of life of oncology patients (Carr et al., 2014; Carr and McCall, 2017).

Since the early 1970s and 1980s, clinicians have reported improved subjective quality of life in patients following administration of both high dose oral and IVC (Cameron and Campbell, 1974; Murata et al., 1982). More recent studies specifically assessing the effects of IVC administration on cancer- and chemotherapy-related quality of life using validated questionnaires have shown decreases in common symptoms, such as fatigue, pain, nausea/vomiting, insomnia and appetite loss, following administration of IVC (Yeom et al., 2007; Takahashi et al., 2012; Stephenson et al., 2013). These studies also demonstrated improvements in overall health and, specifically, enhanced physical, emotional, cognitive, and social functioning. Interestingly, even relatively low IVC doses of 2.5–10 g/infusion provided decreased symptoms and improved quality of life (Yeom et al., 2007; Vollbracht et al., 2011; Kiziltan et al., 2014; Gunes-Bayir and Kiziltan, 2015), suggesting that anti-oxidant/anti-inflammatory functions and/or enzyme cofactor mechanisms of vitamin C may be involved in the quality of life improvements (Carr et al., 2014; Carr and McCall, 2017).

## Q6. What Are the Relevant Mechanisms of Action of IVC?

### I. Generation of Hydrogen Peroxide

Currently, one of the most widely accepted anti-cancer mechanisms proposed for vitamin C is based on its so-called ‘pro-oxidant’ activity (Parrow et al., 2013). However, this terminology can be misleading as vitamin C itself always acts as an antioxidant, through donation of electrons; the ‘pro-oxidant’ action occurs subsequently and is therefore an indirect effect. As such, the term ‘pro-drug’ has been adopted by some researchers (Chen et al., 2005). *In vitro*, vitamin C (in the form of the ascorbate anion) is able to reduce transition metal ions, such as ferric and cupric cations, which are present in buffers or in cell culture media. The reduced transition metal ions are then able to generate hydrogen peroxide through reduction of oxygen to the superoxide radical, which can react with itself to form hydrogen peroxide. However, whether catalytically available transition metal ions are found *in vivo* and can cause enhanced oxidative stress is still a matter of some debate since iron and copper are normally sequestered in transport and storage proteins such as transferrin, ferritin and ceruloplasmin (Carr and Frei, 1999a). Nevertheless, in some pathological situations, such as iron and copper-overload diseases and during tissue injury, free transition metal ions may become more catalytically available (Du et al., 2012). It has also been postulated that transition metal ions are catalytically more available in the extracellular fluid of the tumor microenvironment (Chen et al., 2005).

**FIGURE 1:**
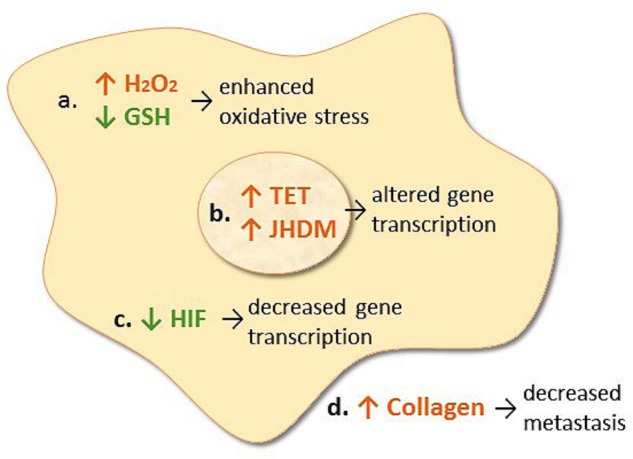
Proposed mechanisms of action of intravenous vitamin C in cancer cells. **(a)** Transition metal ion-dependent generation of hydrogen peroxide (H_2_O_2_) and oxidation of intracellular glutathione (GSH) which causes enhanced oxidative stress and potential cell death. **(b)** Enhances ten-eleven translocation (TET) DNA hydroxylase activity and jumonji histone demethylase (JHDM) activity which alters gene transcription. **(c)** Decreases HIF protein levels which decreases gene transcription. **(d)** Increases collagen synthesis resulting in decreased tumor invasion and metastasis.

*In vitro* studies have shown that addition of high (millimolar) concentrations of vitamin C to cell culture media exhibits differential cytotoxicity toward various cancer cell lines, but not toward normal cultured cells (Chen et al., 2005, 2008). This cytotoxic effect appears to be primarily due to generation of hydrogen peroxide (**Figure 1**), as evidenced by protection via exogenously added catalase (Sestili et al., 1996; Clement et al., 2001). The differential sensitivity of cancer cell lines to vitamin C-generated hydrogen peroxide may reflect their endogenous catalase content (Doskey et al., 2016). Recently, generation of dehydroascorbic acid from vitamin C added to culture media and subsequent uptake of the dehydroascorbic acid into colorectal cancer cells overexpressing glucose transporter 1 was proposed to increase intracellular oxidative stress through oxidation of glutathione (Yun et al., 2015). However, because catalase was not added to the cell cultures, generation of hydrogen peroxide and subsequent oxidative stress due to this reactive oxygen species, rather than dehydroascorbic acid, cannot be ruled out. Furthermore, recent research has shown that addition of vitamin C is more effective than comparable concentrations of enzymatically generated hydrogen peroxide, and addition of non-cytotoxic concentrations of vitamin C with hydrogen peroxide exhibits synergistic effects (Rouleau et al., 2016). This indicates that additional vitamin-dependent anti-tumor mechanisms are occurring.

Pharmacokinetic studies in oncology patients have provided insight into the doses of IVC required to generate high (millimolar) concentrations of vitamin C in plasma (**Table 3**) (Hoffer et al., 2008; Stephenson et al., 2013; Welsh et al., 2013; Nielsen et al., 2015). It is generally believed that plasma vitamin C concentrations > 20 mmol/L are required for hydrogen peroxide generation *in vivo* (Chen et al., 2008). However, it should be noted that hydrogen peroxide generated in the circulation will be rapidly detoxified by antioxidant enzymes present in erythrocytes, including catalase, glutathione peroxidase, and peroxiredoxin-2 (Guemouri et al., 1991; Low et al., 2008). Thus, erythrocytes will act as a sink for hydrogen peroxide generated in the circulation (Zhang et al., 2016). This premise has been confirmed in animal studies whereby low concentrations of hydrogen peroxide were detected in extracellular fluid, but not in blood, following administration of high dose parenteral vitamin C (Chen et al., 2007, 2008).

Administration of high dose parenteral vitamin C (i.e., 1–8 g/kg/d) to murine models has been shown to decrease tumor growth and enhance survival (**Table 4**) (Du et al., 2012; Campbell and Dachs, 2014). The ‘pro-oxidant’ activity of vitamin C is thought to predominate at these higher doses as low levels of hydrogen peroxide and the ascorbyl radical intermediate were detected in the extracellular fluid of rodent models (Chen et al., 2007, 2008). Furthermore, co-administration of high dose thiol antioxidants such as glutathione and *N*-acetyl cysteine inhibited the anti-tumor effect of high-dose vitamin C administration (Chen et al., 2011; Yun et al., 2015). As such, in animal models administered very high dose vitamin C, there is some evidence to suggest that a ‘pro-oxidant’ mechanism is occurring. However, it should be noted that the doses of vitamin C administered to animal models are typically four to eight times higher and more frequent (daily vs. twice a week) than those administered to patients. It should also be noted that most of the animal models used (other than *Gulo* knockout mice and guinea pigs) can also synthesize their own vitamin C. As yet, there is little evidence to indicate that the proposed ‘pro-oxidant’ mechanism is occurring in oncology patients administered IVC (Welsh et al., 2013).

### II. Enzyme Cofactor Activities

Animal studies, particularly in vitamin C-requiring animals such as *Gulo* knockout mice and guinea pigs, have indicated that oral dosing (e.g., 0.3–5 g/L) (Casciari et al., 2005; Gao et al., 2007; Campbell et al., 2015, 2016a; Yang et al., 2017) and low parenteral doses of vitamin C (e.g., 0.1 g/kg/d) (Belin et al., 2009) exhibit comparable decreases in tumor growth to high dose parenteral vitamin C (i.e., 1–8 g/kg/d; **Table 4**). Since hydrogen peroxide is not detected following oral or low dose IVC administration (Chen et al., 2007), this suggests possible tumor growth inhibition mechanisms other than hydrogen peroxide generation are likely operating in these situations. Numerous cell culture and pre-clinical studies have shown regulatory effects of vitamin C administration on various transcription factors and cell signaling pathways, with subsequent effects on cell cycle, angiogenesis, and cell death pathways (Parrow et al., 2013). Analysis of tissues from various animal models has indicated regulation of numerous genes and their products following administration of vitamin C (Gao et al., 2007; Belin et al., 2009; Park et al., 2009; Yeom et al., 2009; Lee et al., 2012; Ma et al., 2014; Campbell et al., 2015, 2016a,b). Thus, it appears that vitamin C administration, particularly at lower doses, has gene-regulatory effects.

Vitamin C is a cofactor for a family of metalloenzymes with pleotropic biosynthetic and gene regulatory roles (Du et al., 2012). It has long been known to be a cofactor for the three hydroxylase enzymes essential for the stabilization of collagen tertiary structure (Englard and Seifter, 1986). Research has shown increased collagen encapsulation of tumors in *Gulo* knockout mice with melanoma and breast tumors following supplementation with low dose oral vitamin C (0.15 g/L, **Figure 1**) (Cha et al., 2011, 2013). Decreased metastases were also observed in these models. This work has been confirmed recently in a pancreatic cancer model (Polireddy et al., 2017). Increased levels of collagen were detected in tumor stroma of vitamin C-treated mice and this was associated with decreased tumor invasion. Resected tumors from pancreatic cancer patients treated with IVC also exhibited increased collagen content (Polireddy et al., 2017). Since enhanced collagen mRNA levels were also observed in the tumors from vitamin C-treated mice, this suggests that gene regulatory mechanisms are also involved.

In the early 2000s, vitamin C was demonstrated to regulate the transcription factor hypoxia inducible factor-1α (HIF-1α) (Hirsila et al., 2003; Koivunen et al., 2004; Hirota and Semenza, 2005). HIF-1α is a constitutively expressed transcription factor which regulates numerous pro-survival genes. Under normoxic conditions, HIF-1α is downregulated via hydroxylase-mediated modifications which prevents coactivator binding and targets HIF for proteosomal degradation. In the hypoxic core of solid tumors HIF-1α is upregulated due to the absence of substrates and cofactors required for hydroxylase-dependent downregulation. Vitamin C is a cofactor for the HIF hydroxylases (Kuiper and Vissers, 2014). Animal studies have shown downregulation of HIF-1α and downstream pro-survival proteins (e.g., glucose transporter 1, vascular epithelial growth factor, and carbonic anhydrase) following administration of oral or parenteral vitamin C (**Figure 1**) (Campbell et al., 2015, 2016a,b). In human colorectal tumors and other tumors there was an inverse association between tumor vitamin C levels and expression of HIF and related downstream proteins (Kuiper et al., 2010, 2014a; Jozwiak et al., 2017), and enhanced disease-free survival was observed in patients who had higher vitamin C levels in their tumors (Kuiper et al., 2014a).

Recent research has uncovered a role for vitamin C in epigenetic regulation via acting as a cofactor for DNA and histone demethylases which belong to the same family of enzymes as the collagen and HIF hydroxylases (Camarena and Wang, 2016; Gillberg et al., 2017). Vitamin C acts as a cofactor for the ten-eleven translocation (TET) dioxygenases which hydroxylate methylated cytosine moieties in DNA (Blaschke et al., 2013; Minor et al., 2013; Yin et al., 2013). The hydroxymethylcytosine mark can be further oxidized and subsequently removed through both active and passive DNA repair mechanisms, but may also represent an epigenetic mark in its own right (Camarena and Wang, 2016). It is noteworthy that a decrease in DNA hydroxymethylation has been observed in cancer cells and tumors (Haffner et al., 2011; Kudo et al., 2012; Lian et al., 2012; Kroeze et al., 2015). Recent research has indicated that vitamin C can modulate the epigenome of leukemia cells and regulate hematopoietic stem cell function in a TET-dependent fashion, suppressing leukemia progression in pre-clinical models (**Figure 1**) (Agathocleous et al., 2017; Cimmino et al., 2017; Mingay et al., 2017; Zhao et al., 2018). Research has also indicated that vitamin C treatment increases hydroxymethylation in lymphoma and melanoma cells, and causes a decrease in tumor-cell invasiveness and clonogenic growth (Gustafson et al., 2015; Shenoy et al., 2017). Thus, epigenetic mechanisms may be involved in the attenuated metastasis that has been observed in animal models and patients following administration of vitamin C (Padayatty et al., 2006; Pollard et al., 2010; Cha et al., 2013; Polireddy et al., 2017).

Vitamin C is also a cofactor for several Jumonji C domain-containing histone demethylases (JHDM) that catalyze histone demethylation (Camarena and Wang, 2016). Methylation of lysine and arginine residues on histones is closely associated with activation or silencing of transcription. JHDM can demethylate mono-, di-, and trimethylated histone lysine and arginine residues (Klose et al., 2006). Vitamin C is required for optimal catalytic activity and demethylation by JHDM (Tsukada et al., 2006). The involvement of vitamin C in JHDM-dependent histone demethylation was confirmed in somatic cell reprogramming and T-cell maturation (Wang et al., 2011; Manning et al., 2013; Ebata et al., 2017). Thus, it appears that vitamin C can regulate the epigenome via acting as a cofactor for both DNA and histone demethylases. Due to the multitude of genes regulated through both DNA and histone demethylation, it is likely that epigenomic regulation by vitamin C may play a major role in its pleiotropic health promoting and disease modifying effects. Continuing research in this field will no doubt reveal exciting insights and treatment possibilities.

### III. Antioxidant and Anti-inflammatory Activities

It has been suggested that oxidative stress, chronic inflammation, and cancer are closely linked (Reuter et al., 2010). Oxidative stress can activate a variety of transcription factors, leading to the expression of hundreds of different genes, including those of pro-inflammatory cytokines. Vitamin C is a potent antioxidant both in plasma and within cells due to its ability to scavenge a wide range of reactive oxygen species, thereby protecting important biomolecules from oxidative damage (Carr and Frei, 1999a). Patients with cancer tend to have elevated markers of oxidative stress, such as malondialdehyde (Hunnisett et al., 1995; Torun et al., 1995; Mahdavi et al., 2009; Sharma et al., 2009; Mehdi et al., 2013). An early study in healthy volunteers administered low dose IVC showed a decrease in lipid oxidation biomarkers (Muhlhofer et al., 2004). More recently, administration of high dose IVC to patients with pancreatic cancer produced a decrease in F_2_-isoprostanes, a marker of systemic oxidative stress (Welsh et al., 2013), suggesting a systemic antioxidant effect of IVC. Detection of ascorbyl radicals in the blood and extracellular fluid of animal models has been used as evidence for a ‘pro-oxidant’ role for vitamin C, i.e., they are an intermediate in the reduction of transition metal ions (Chen et al., 2007, 2008). Although very low (nanomolar) levels of ascorbyl radical were detected in the circulation of patients in the human intervention study (Welsh et al., 2013), these radicals can also be formed through vitamin C’s oxidant scavenging function (Carr and Frei, 1999a). Thus, detection of low level ascorbyl radicals, which parallels ascorbate concentrations, could instead be indicative of oxidant scavenging, i.e., an antioxidant, role of vitamin C (Muhlhofer et al., 2004).

Elevated markers of inflammation, such as C-reactive protein and various cytokines, have been reported in oncology patients (Mayland et al., 2005; Mikirova et al., 2012, 2013, 2016; Nannya et al., 2014). Vitamin C exhibits anti-inflammatory functions via modulating cytokine levels (Carr and Maggini, 2017) and animal models of melanoma and breast cancer have indicated decreased pro-inflammatory cytokine (interleukin-6, interleukin-1) levels following vitamin C administration (Cha et al., 2011, 2013). In patients with various cancers, administration of 25–50 g IVC decreased a number of different inflammatory mediators (such as C-reactive protein) and pro-inflammatory cytokines (Mikirova et al., 2012, 2016). Because oxidants can enhance inflammation, it is not clear if the cytokine-modulatory effects of vitamin C are due to its oxidant scavenging function or its gene regulatory cofactor functions (Song et al., 2017). It is noteworthy that patients with higher levels of inflammation also appear to have a higher requirement for vitamin C (Mikirova et al., 2013).

## Q7. What Are the Optimal Doses, Frequency, and Duration of IVC Therapy?

Intravenous vitamin C has been used for decades by health care professionals with very little consistency in the dose, frequency, or duration of use. A survey of complementary and alternative medicine practitioners showed an average dose of 28 g/infusion (range of 1–200 g/infusion), a frequency of approximately twice a week (range of 1–7 times per week), and about 19 treatments per patient (range of 1–80 treatments) (Padayatty et al., 2010). Because of the prevailing ‘pro-oxidant’ paradigm of IVC, it is generally believed that ‘more is better’ and doses as high as 200 g/infusion have been administered to oncology patients (**Table 5**) (Padayatty et al., 2010; Stephenson et al., 2013). Lower IVC doses of 2.5–10 g/infusion have been shown to decrease common cancer- and chemotherapy-related symptoms and improve health-related quality of life (Carr et al., 2014).

Although the ‘pro-oxidant’ activity of IVC has yet to be conclusively demonstrated in humans, there may be alternate rationales for administering high dose IVC. Solid tumors exhibit dysregulated blood supply which limits diffusion of oxygen and other nutrients to the core of the tumors. Utilizing a well-established multicell-layered, three-dimensional pharmacokinetic model, Kuiper et al. (2014b) measured vitamin C diffusion and transport parameters through dense tissue. The investigators demonstrated that supra-physiological plasma concentrations (i.e., up to 500 μmol/L) were required to achieve effective delivery of vitamin C to poorly vascularized tumor tissue. Enhanced delivery of vitamin C to the hypoxic core of solid tumors would facilitate down-regulation of the HIF-driven hypoxic response (Kuiper and Vissers, 2014). The efficacy of IVC against metastatic tumors could also be due, in part, to the generally smaller size of metastases allowing better diffusion of vitamin C into the tumor, thus facilitating its various gene-regulatory mechanisms. Uptake of vitamin C into tumor cells may also be dependent upon vitamin C transporter (SVCT2) status and polymorphisms, which is an area that warrants further investigation (Wang C. et al., 2017; Wohlrab et al., 2017).

Preclinical studies indicate that a single infusion of vitamin C is not as effective as multiple infusions and a higher frequency of administration appears to be more beneficial (Takemura et al., 2010; Campbell et al., 2016b). For example, daily intraperitoneal injections of vitamin C slowed tumor growth in the mice and downregulated HIF-1α and downstream gene products to a greater extent than injections every other day (Campbell et al., 2016b). A trial of castration-resistant prostate cancer patients who were administered only one 60 g infusion per week did not show diminished PSA levels or disease remission after 12 weeks (Nielsen et al., 2017). However, daily administration of high dose IVC to rodents with implanted prostate tumor cells showed decreased tumor growth and lung metastases (Pollard et al., 2010). Although continuous vitamin C infusion has been piloted (Riordan et al., 2005), this is usually only practical if the patients are hospitalized. However, because IVC solutions are remarkably stable, even at ambient temperature and in the light (Carr et al., 2018), it might be possible to utilize continuous infusion bottles typically used for home intravenous antibiotic administration. However, since intermittent infusions allow for high peak plasma concentrations (de Grooth et al., 2018), there is debate as to whether continuous infusion is better than intermittent infusions over a longer period (Cameron, 1991; Gonzalez et al., 2012). As mentioned above, high peak concentrations may be required to facilitate uptake of vitamin C into solid tumors.

## Conclusion

• Do oncology patients have compromised vitamin C status? Yes, studies consistently show that patients with cancer have lower mean circulating vitamin C levels than healthy volunteers. These patients also exhibit higher rates of hypovitaminosis C and deficiency. Furthermore, chemotherapy can impact negatively on the vitamin C status of oncology patients. Because of vitamin C’s supportive functions in the body, increasing the vitamin C status of oncology patients is likely to be of benefit.
• Is IV the optimal route for vitamin C administration? Yes, IV administration of vitamin C can provide significantly higher peak plasma concentrations because it bypasses the regulated intestinal uptake of oral vitamin C. These higher concentrations are believed to be required for some of the proposed anti-cancer mechanisms of vitamin C and may also enhance diffusion of the vitamin into the hypoxic core of solid tumors.
• Is IVC safe? Yes, IVC is remarkably safe, considering the massive (>75 g) doses that are often administered. However, there are several currently known situations where caution is warranted. These include patients with impaired renal function due to their inability to adequately clear high IVC doses from circulation, and patients with G6PD deficiency due to inability to detoxify oxidative stress generated by high dose IVC administration. Caution is also required for patients requiring regular glucose monitoring due to the potential for IVC to interfere with point-of-care glucose monitors.
• Does IVC interfere with chemotherapy or radiotherapy? Clinical trials indicate that IVC does not adversely interfere with chemotherapy and pre-clinical studies indicate that it may in fact act synergistically in combination with different chemotherapeutic agents. There is as yet limited research around interference with radiotherapy, with conflicting results likely due to the timing of the interventions.
• Does IVC decrease the toxic side effects of chemotherapy and improve quality of life? Both pre-clinical and clinical studies indicate that IVC can decrease the off-target toxicity of chemotherapeutic agents, likely through its antioxidant and anti-inflammatory activities, without affecting the anti-cancer activities of the chemotherapeutic agents. The reduction in specific chemotherapy-related side-effects results in an overall improvement in the health-related quality of life of oncology patients.
• What are the relevant mechanisms of action of IVC? A number of plausible anti-cancer mechanisms have been proposed, such as indirect generation of hydrogen peroxide, enzyme cofactor activities (e.g., collagen synthesis, HIF hypoxic response regulation, TET and JHDM epigenetic regulation), as well as antioxidant and anti-inflammatory functions. Different cancers likely respond differently to IVC therapy depending upon their underlying mechanisms. Thus, future work should focus on tailoring IVC regimens to specific cancers or cancer subtypes, e.g., hematological cancers that are driven specifically by TET mutations may respond more readily to IVC therapy.
• What are the optimal doses, frequency, and duration of IVC therapy? Although these are the most relevant questions clinically, there is still little consensus as to how much, how often and for how long to administer IVC to oncology patients. The different proposed mechanisms of action provide some insight into dosing, with higher doses (>50 g/d) being required for some anti-cancer mechanisms, and lower doses (≤10 g/d) being sufficient for decreasing symptoms and improving quality of life. Pre-clinical studies indicate that more frequent dosing exhibits enhanced efficacy. However, depending on the underlying mechanisms involved, it is possible that anti-tumor activity may require long term treatment and follow-up, e.g., over years rather than just the few weeks or months of most clinical trials. It is unlikely that future large scale IVC RCTs will be carried out due to the prohibitive costs. Nevertheless, smaller scale studies, if well designed, have the potential to contribute relevant and translatable findings to inform good clinical practice.

## Author Contributions

AC conceived and wrote the review. JC contributed clinical input.

## Conflict of Interest Statement

The authors declare that the research was conducted in the absence of any commercial or financial relationships that could be construed as a potential conflict of interest.
